# Tension Band Wiring in the Treatment of Extensor Carpi Radialis Longus Avulsion Fractures at the Base of the Second Metacarpal: A Case Report and Literature Review

**DOI:** 10.7759/cureus.67677

**Published:** 2024-08-24

**Authors:** Mustafa Özyildiran

**Affiliations:** 1 Department of Orthopedics and Traumatology, Afyonkarahisar Sandıklı State Hospital, Afyonkarahisar, TUR

**Keywords:** tension band wiring, ecrl, extensor carpi radialis longus, second metacarpal, avulsion fracture

## Abstract

An avulsion fracture of the second metacarpal is a rare injury often resulting from resisted wrist hyperflexion, and there is no consensus on the optimal treatment. A review of the literature reveals 20 articles documenting 25 cases of similar injuries. Of these, nine cases were initially managed conservatively, while 16 were treated surgically. Among the nine conservative cases, five (55.6%) required late surgical intervention due to unsuccessful initial treatment. In contrast, none of the 16 surgically treated cases reported poor clinical outcomes. This case involves a 23-year-old male with an extensor carpi radialis longus avulsion fracture at the base of the second metacarpal, treated with open reduction and tension band wiring. The patient achieved favorable postoperative results. In other reported cases, fixation methods included Kirschner wires, screws, or miniplates. To our knowledge, this is the first case using tension band wiring for this type of injury.

## Introduction

Avulsion fractures at the base of the second metacarpal involving the insertion of the extensor carpi radialis longus (ECRL) tendon are rare injuries [1-3]. These injuries typically result from a violent blow to the hand, with forced volar flexion of the wrist being a common mechanism. The dorsal and volar capsuloligamentous complex, along with the bony architecture, restricts movement at the second carpometacarpal joint. Due to the rigidity at the base of the metacarpal, forced hyperflexion often causes avulsion of the ECRL tendon at its bony attachment rather than dislocation [4,5]. These fractures are frequently challenging to detect on standard radiographs and can be difficult to diagnose accurately at initial presentation, leading to missed diagnoses [5,6]. Clinical suspicion and CT are valuable for preventing delayed diagnosis [7,8].

There is no consensus on the optimal treatment method, with options ranging from conservative approaches to open surgical procedures [1-10]. To date, open reduction and tension band wiring for ECRL avulsion fractures have not been reported in the literature. This case report describes the surgical treatment of such an injury using tension band wiring and provides a review of the existing literature.

## Case presentation

**Figure 1:**
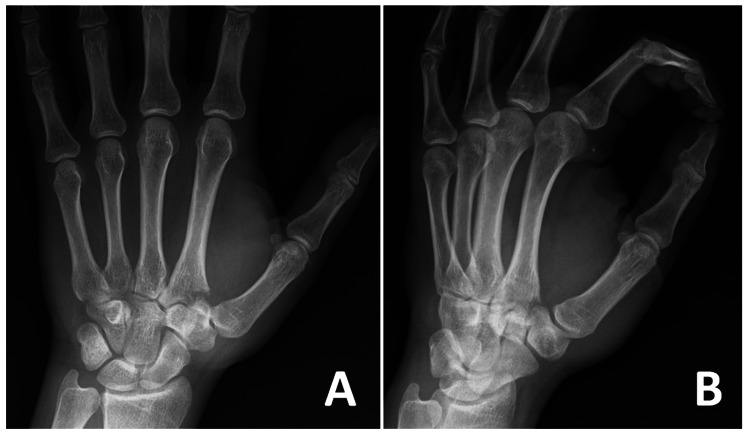
Preoperative (A) posteroanterior and (B) oblique hand radiographs

A 23-year-old right-hand dominant man presented to the emergency department with pain and swelling in his left hand following a punch during a fight. Due to intoxication, he was unsure of the exact mechanism of injury but noted that his left hand had been forcibly flexed while punching. Physical examination revealed swelling and tenderness at the base of the second metacarpal, with extremely painful and limited wrist extension. The injured extremity was neurovascularly intact, and the skin was undamaged. No other injuries were noted except for the left hand. Radiographs showed an avulsion fracture at the base of the second metacarpal (Figure 1).

**Figure 2:**
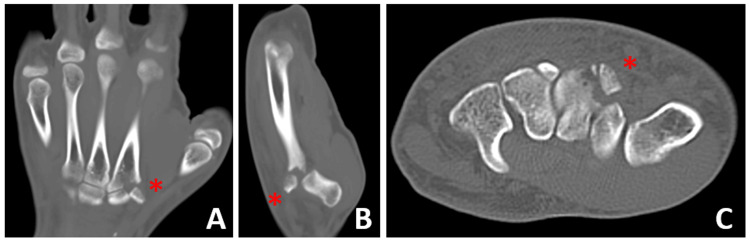
(A) Coronal, (B) sagittal, and (C) axial CT scans showing ECRL avulsion fracture at the base of the second metacarpal bone Red asterisks indicate the avulsed fragment. ECRL, extensor carpi radialis longus

CT scans revealed proximal migration and a 90-degree rotation of the avulsed fragment (Figure 2). The patient was initially immobilized in a short arm splint for pain relief, and an open reduction was planned.

A supraclavicular brachial plexus block was administered, and the procedure was performed under a tourniquet. A dorsal longitudinal skin incision was made over the base of the second metacarpal. The extensor pollicis longus (EPL) tendon and branches of the superficial radial nerve were identified and protected. The ECRL tendon was intact. A small avulsed bone fragment attached to the ECRL tendon was found, displaced proximally, and rotated. The fragment was anatomically reduced with the wrist extended and stabilized with two 1.0 mm Kirschner wires, placed longitudinally and crossing the anterior cortex.

**Figure 3:**
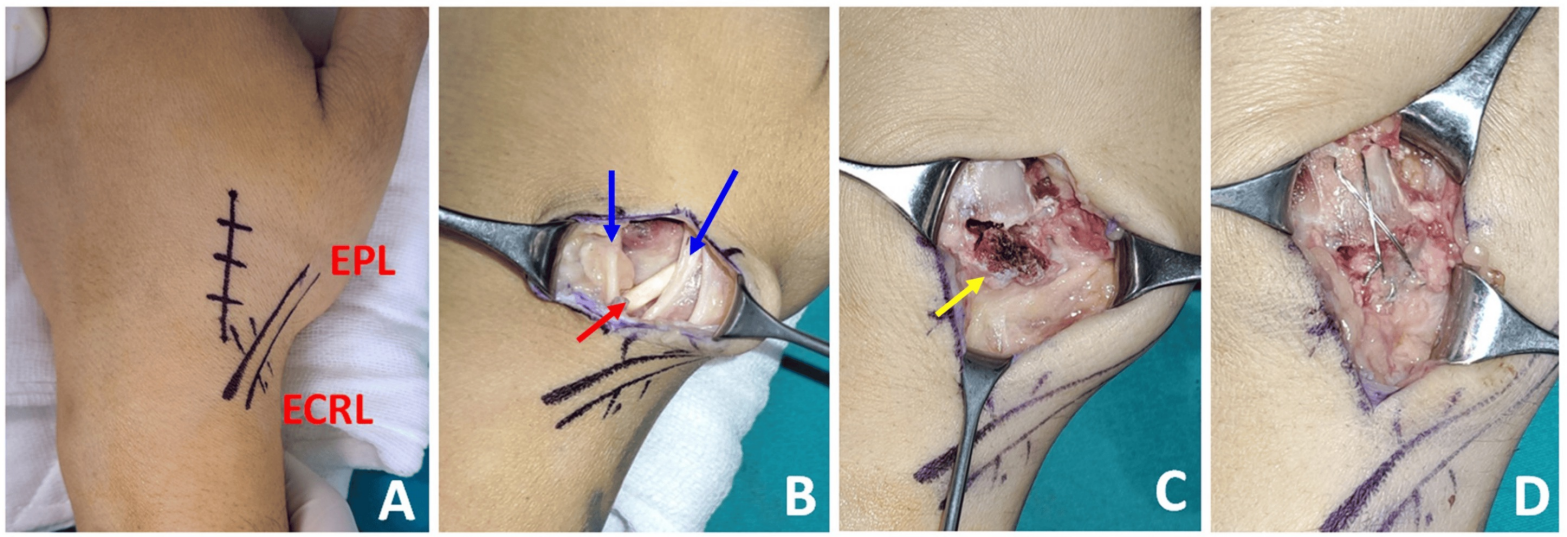
Intraoperative photographs of tension band wiring (A) Projections of the ECRL and EPL were drawn with a marker pen. (B) A dorsal longitudinal skin incision was made over the base of the second metacarpal bone. EPL and the branches of the superficial radial nerve were identified and protected; blue arrows indicate the nerve branches, and the red arrow shows the EPL tendon. (C) The avulsion fracture at the insertion of the ECRL tendon was identified, with the yellow arrow marking the avulsed fragment. (D) After anatomical reduction, fixation was performed using the tension band technique. ECRL, extensor carpi radialis longus; EPL, extensor pollicis longus

**Figure 4:**
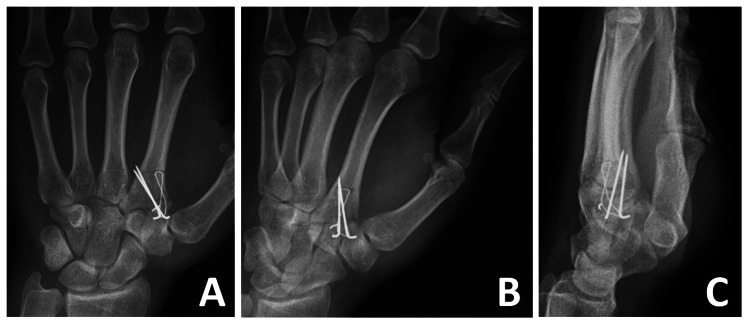
Postoperative (A) posteroanterior, (B) oblique, and (C) lateral hand radiographs

Given the small size of the avulsed fragment, tension band wiring was chosen over screw fixation. A 1.0 mm K-wire was used to drill a bone tunnel in the shaft of the second metacarpal. A 26-gauge stainless steel wire, doubled for increased thickness, was used for tension band wiring. The wire was passed through the bone tunnel and looped in a figure-eight pattern to secure the fracture (Figure 3, Figure 4).

Postoperatively, the wrist was immobilized in a short arm splint with the wrist extended 30° to control pain and edema. The splint was removed two weeks after surgery, and wrist range of motion exercises were initiated. Hand therapy began in the fourth week. By the third postoperative month, the patient had normal wrist strength and a full range of wrist movement. The procedure was well tolerated, with no adverse outcomes or implant-related discomfort.

At eight months postoperatively, the hardware was removed. During the surgery, it was noted that the avulsion fracture had completely healed and bony union had been achieved. Three months after implant removal, clinical scores were excellent, with a Disabilities of the Arm, Shoulder and Hand (DASH) score of 3.3 (range: 0-100). Grip strength in the operated hand was 40 kg compared to 45 kg in the contralateral hand (measured with a JAMAR dynamometer).

## Discussion

A literature search was performed using the Medline/PubMed database with the keywords “extensor carpi radialis longus,” “ECRL,” “second metacarpal,” and “avulsion fracture.” The review included cases of ECRL avulsion fractures at the base of the second metacarpal, excluding those with only ECRL tendon ruptures without associated avulsion fractures. The search identified 20 published case reports covering 25 cases of ECRL avulsion fractures. Of these, nine cases were initially treated conservatively, while 16 were treated surgically (Table 1).

**Table 1:** Existing case reports in the literature on avulsion fractures of the second metacarpal ECRB, extensor carpi radialis brevis; ECRL, extensor carpi radialis longus; EPL, extensor pollicis longus

Author and year	N	Treatment	Clinical result	
				McInnes (1947) [11]	1	Open reduction and K-wire fixation	No information	
DeLee (1979) [12]	1	Open reduction and K-wire fixation	Good	
Sadr and Lalehzarian (1987) [13]	1	Excision of the bony fragment and side-to-side suturing of the ECRL to ECRB	Weakness in the wrist extensors and the grip strength	
			Delayed surgery due to initial misdiagnosis	
Treble and Arif (1987) [4]	1	Open reduction and K-wire fixation	No information	
Crichlow and Hoskinson (1988) [14]	1	Conservative	Good	
	2	Conservative	Good	
	3	Initial conservative	Excision of the bony prominence due to unacceptable cosmetic appearance and pain	
Cassell and Vidal (1996) [6]	1	Open reduction and internal fixation	No information	
Jessa and Hodge (1997) [15]	1	Conservative	No information	
	2	Conservative	No information	
Takami et al. (1998) [16]	1	Initial conservative	Secondary surgery (due to fragment dislocation and pain)	
			Open reduction and K-wire fixation	
Boles and Durbin (1999) [17]	1	Open reduction and screw fixation of the bone fragment	Good	
		Suture anchor repair for ECRL and ECRB		
Jena et al. 2001 [18]	1	Initial conservative	Secondary surgery (due to displacement of the fracture fragment)	
			Open reduction and K-wire fixation	
Ishida et al. (2006) [19]	1	Open reduction and screw fixation of the bone fragment	Good	
		EPL tendon repair with end-to-end suture		
Punjabi and Leong (2007) [2]	1	Open reduction and screw fixation of the bone fragment	Good	
		Suture anchor repair for ECRL		
Kim et al. (2008) [20]	1	Open reduction and miniplate osteosynthesis	Good	
	2	Open reduction and miniplate osteosynthesis	Good	
	3	Open reduction and miniplate osteosynthesis	Good	
Clark et al. (2008) [3]	1	Open reduction and screw fixation	Good	
Gurich et al. (2013) [5]	1	Initial conservative	Secondary surgery (due to dorsal wrist pain with limited wrist extension)	
			Suture anchor fixation of the ECRB and tenodesis of the retracted ECRL to the ECRB	
Robert et al. (2014) [1]	1	Open reduction and screw fixation	Good	
Cattelan and Meier (2015) [8]	1	Open reduction and miniplate osteosynthesis	Good	
Najefi et al. (2016) [9]	1	Open reduction and screw fixation	Good	
Alnaif et al. (2018) [7]	1	Open reduction and screw fixation of the bone fragment	Good	
		Suture anchor repair for ECRL		
Beutel and Melamed (2019) [10]	1	Initial conservative	Secondary surgery (due to extension weakness and ulnar deviated posture)	
			Lengthening of the ECRL and ECRB tendons and suture anchor fixation	
Our case (2024)	1	Open reduction and tension band wiring	Good	

The nine cases initially managed conservatively originated from six reports. Of these, two cases showed favorable outcomes, while two lacked outcome information, and five ultimately required late surgical intervention (Table 1). Late surgical intervention was necessary due to issues such as fracture fragment displacement, limited wrist extension, and dorsal wrist pain [5,10,14,16,18]. Beutel et al. noted that chronic cases might develop ulnar-deviated wrist posture due to ECRL avulsion. Their report described a case where surgical intervention was performed five months post-injury on a patient initially treated with cast immobilization due to increased ulnar deviation with active wrist extension and grip strength weakness [10]. Tendon retraction in chronic injuries can complicate late surgical intervention [5,10].

In ECRL avulsion fractures, potential concomitant damage to the EPL tendon should be considered. Cassel [6] and Ishida [19] reported EPL rupture associated with avulsed metacarpal fragments. They performed open reduction and internal fixation (ORIF) of the second metacarpal base and repaired the EPL tendon with end-to-end sutures. Although not documented, EPL rupture due to fragment dislocation post-conservative treatment is a theoretical risk associated with conservative management.

Most authors support ORIF. Among the 25 reported cases, 16 were treated surgically (Table 1), with no poor outcomes reported. Sadr and Lalehzarian [13] observed ECRL retraction due to late surgical intervention and could not reattach the ECRL to its insertion. They performed bony fragment excision and side-to-side suturing of ECRL to ECRB. While the patient was satisfied, the clinical result was less favorable due to the delayed intervention [13]. Information on three cases treated with ORIF was unavailable [4,6,11]. Nevertheless, 12 out of 16 (75.0%) surgically treated patients had good outcomes (Table 1) [1-3,7-9,12,17,19,20].

Fixation methods for ORIF included Kirschner wires, screws, or miniplates, with no consensus on the optimal technique. Factors such as the surgeon’s experience, fracture pattern, and implant availability influence the choice. For instance, McInnes [11], DeLee [12], and Treble and Arif [4] used K-wire fixation, while Kim [20] and Cattelan and Meier [8] favored miniplate osteosynthesis. Other authors opted for screw fixation, using different types: Najefi et al. [9] (headless cannulated screw, 26 mm mini Acutrak), Robert et al. [1] (2.3 mm cannulated compression screw), Clark et al. [3] (2.0 and 1.7 mm screws), and Punjabi and Leong [2] (1.3 mm hand modular set screw).

In this case, the very small size of the avulsed fragment led us to choose tension band wiring over screw fixation. Tension band wiring is a technique suited for fractures caused by tension or bending forces. It involves placing a stabilizing construct along the tension side of a fracture, typically with steel wires twisted in a figure-eight pattern. This method converts tensile forces into compressive forces, providing stability. Tension band wiring is effective for various fractures, including olecranon, patella, malleolus, and avulsion fractures of the base of the proximal phalanx. To our knowledge, this is the first report of using tension band wiring for ECRL avulsion fractures. The technique offers several advantages: mechanical stability, fracture site compression, early mobilization, and cost-effectiveness. For small avulsed fragments, screw fixation risks bone crumbling, and using two screws might not be feasible due to size constraints, while a single screw may not provide adequate rotational stability. Thus, tension band wiring is an advantageous method for such cases.

## Conclusions

Avulsion fractures at the base of the second metacarpal are uncommon, but ORIF typically yields satisfactory outcomes. This case study presents the successful management of an ECRL avulsion fracture at the base of the second metacarpal in a 23-year-old male patient using open reduction and tension band wiring. The postoperative results were favorable. Notably, this is the first report to describe tension band wiring for these fractures. This technique offers advantages over other fixation methods, particularly for fractures with very small avulsed fragments. Tension band wiring should be considered as a viable alternative when planning surgical intervention.
